# Genome-Wide Analysis of the Membrane Attack Complex and Perforin Genes and Their Expression Pattern under Stress in the Solanaceae

**DOI:** 10.3390/ijms241713193

**Published:** 2023-08-25

**Authors:** Sirui Ma, Yixian Guo, Di Liu, Xue Zhang, Jihong Guo, Tianyi Zhang, Lin Lai, Yi Li, Qinfang Chen, Lujun Yu

**Affiliations:** State Key Laboratory of Biocontrol, Guangdong Key Laboratory of Plant Resources, School of Life Sciences, Sun Yat-sen University, Guangzhou 510275, China; masr469@163.com (S.M.); guoyx67@mail2.sysu.edu.cn (Y.G.); liud47@mail2.sysu.edu.cn (D.L.); cherxuer@163.com (X.Z.); guojihong601@163.com (J.G.); skybruce091@sina.com (T.Z.); lailin1014@163.com (L.L.); liyi253@mail2.sysu.edu.cn (Y.L.); chenqf3@mail.sysu.edu.cn (Q.C.)

**Keywords:** *MACPF*, evolution, expression pattern, Solanaceae

## Abstract

The Membrane Attack Complex and Perforin (MACPF) proteins play a crucial role in plant development and adaptation to environmental stresses. Heretofore, few *MACPF* genes have been functionally identified, leaving gaps in our understanding of *MACPF* genes in other plants, particularly in the Solanaceae family, which includes economically and culturally significant species, such as tomato, potato, and pepper. In this study, we have identified 26 *MACPF* genes in three Solanaceae species and in the water lily, which serves as the base group for angiosperms. Phylogenetic analysis indicates that angiosperm *MACPF* genes could be categorized into three distinct groups, with another moss and spikemoss lineage-specific group, which is further supported by the examination of gene structures and domain or motif organizations. Through inter-genome collinearity analysis, it is determined that there are 12 orthologous *SolMACPF* gene pairs. The expansion of *SolMACPF* genes is primarily attributed to dispersed duplications, with purifying selection identified as the principal driving force in their evolutionary process, as indicated by the ω values. Furthermore, the analysis of expression patterns revealed that Solanaceae genes are preferentially expressed in reproductive tissues and regulated by various environmental stimuli, particularly induced by submergence. Taken together, these findings offer valuable insights into and a fresh perspective on the evolution and function of *SolMACPF* genes, thereby establishing a foundation for further investigations into their phenotypic and functional characteristics.

## 1. Introduction

The Membrane Attack Complex and Perforin (MACPF) protein encompasses the membrane attack complex (MAC) and perforin proteins, which are present in various organisms such as fungi, plants, and animals [1,2,3]. These proteins play a crucial role in the organisms defense against bacterial and viral infections, contributing to immune response and cell lysis [4]. The MACPF protein has the ability to create pores in cellular membranes [5]. Its structure is closely associated with cholesterol-dependent cytolysins and the complement system, which includes proteins such as C6, C7, C8α, C8β, and C9 [6,7,8,9]. In recent times, the resolution of several MACPF protein structures has revealed that the MACPF domain of complement proteins undergoes significant structural rearrangements during the formation of pores. These rearrangements involve a conserved core fold, while the C-terminal domains exhibit variability, thereby influencing interactions with other proteins [6,10,11,12,13].

Over the past two decades, extensive research has demonstrated the crucial involvement of certain MACPF proteins in the development and immune responses of organisms, particularly in the animal kingdom [2,12,14,15,16]. However, the understanding of regulatory mechanisms governing *MACPF* genes in plants has progressed at a considerably slower pace compared to animals.

However, the precise functions and underlying molecular mechanisms of MACPF proteins in plants are poorly understood. To date, only three MACPF proteins, namely necrotic spotted lesions 1 (NSL1, At1g28380), CONSTlTUTIVELY ACTIVATED CELL DEATH 1 (CAD1, also referred to as NECROTIC SPOTTED LESIONS 2 or NSL2), and MACP2 in *Arabidopsis thaliana*, have been characterized and shown to play roles in regulating plant immunity and salicylic acid (SA)-mediated defense signaling pathways, as well as in restricting fungal invasion through the transport of phenolic compounds. Furthermore, the involvement of *MACPF* genes in plant stress responses has only been investigated in three *Arabidopsis thaliana* genes [15,17,18,19,20,21,22,23]. Arabidopsis NSL1 is a crucial factor in the inhibition of cell death and defense responses, whose mutant displays impaired growth and development, along with the emergence of spotted necrotic lesions on leaves, even in the absence of pathogens [17]. Additionally, the *nsl1* mutant exhibits elevated levels of salicylic acid (SA), which is a consequence of the Trp-derived activation of the SA pathway, implying that NSL1 acts as a negative regulator of cell death and is indispensable for its prevention [19]. Furthermore, it has been observed that CAD1 is localized in both the cytosol and plasma membrane [22], which is also found to have a negative regulatory effect on the expression of SA-related defense genes and the activation of systemic acquired resistance (SAR) through hypersensitive response (HR) [15,18,21]. Additionally, CAD1 has been implicated in the regulation of autoimmunity by interacting with ENHANCED DISEASE SUSCEPTIBILITY 1 (EDS1), a key protein in the plant immune system [22]. Another protein, MACP2 (At4g24290), has also been shown to induce programmed cell death, enhance bacterial pathogen resistance, and suppress resistance against necrotrophic fungal pathogens, which is achieved by stimulating the biosynthesis of indole glucosinolates and the SA pathway [23]. Furthermore, the regulation of mRNA isoforms of MACP2 was found to be influenced by alternative splicing and exposure to pathogen attack, which distinguishes it from other *MACPF* genes in Arabidopsis [23]. These findings suggest that *MACPF* plays a significant role in the plant’s response to environmental stresses, and further research in other plant species, apart from Arabidopsis, is warranted. To date, several *MACPF* genes have been identified at the genome level in Arabidopsis, cotton, moss, spikemoss, and Poaceae [2,19,23]. Notably, the *MACPF* gene has not been identified in algae, with its first occurrence in moss *P. patens*, which could be related to the plant landing [2].

In addition, the plant *MACPF* gene family has been categorized into four groups, with Group IV being identified as the specific gene family for moss and spikemoss [2]. However, the distribution of *MACPF* genes in other plants, particularly in economically significant crops, like Solanaceae, remains unexplored despite the availability of well-sequenced and annotated species, such as tomato, potato, and pepper. Additionally, the recent sequencing of water lily (*Nymphaea colorata*), which serves as the basal group of angiosperms [24], offers a valuable opportunity to investigate the evolutionary dynamics of the *MACPF* gene family at a more comprehensive level. The systematic and evolution study will fulfill the functional studies of the *MACPF* genes in Solanaceae species.

Here, the genomic level of *MACPF* genes was identified in both Solanaceae and water lily species, followed by subsequent analyses of their evolution and expression profiles. The findings will offer novel insights into the evolution and function of *SolMACPF* genes, serving as a foundation for future investigation.

## 2. Results

### 2.1. Identification of MACPF Members in Solanaceae Species Genomes

In our previous research, we successfully identified *MACPF* genes in 15 plant genomes, encompassing various species, such as green algae, moss, spikemoss, eudicots, and six Poaceae species, which indicated a potential correlation between MACPF proteins and plant colonization on land [2]. Building on these findings, we have now applied the same identification criteria to filter *MACPF* genes in the genomes of Solanaceae species. Our analysis revealed the presence of six, seven, and six *MACPF* genes in *Capsicum annuum*, *Solanum lycopersicum*, and *Solanum tuberosum*, respectively. The longest transcripts of the *MACPF* genes were chosen, which were subsequently named according to the genome location (Table S1). Furthermore, we have successfully identified the 7 *MACPF* genes in *Nymphaea colorata*, which belong to the basal group of seed plants.

The predicted Solanaceae MACPF proteins exhibited a comparable size range of 565 to 716 amino acids, as shown in Table S1, which is consistent with the MACPF size observed in Arabidopsis and rice. Additionally, the physical and chemical properties of these proteins were calculated using the EXPASY website (http://www.expasy.org, accessed on 18 August 2023). The findings revealed that the molecular weight (MW) ranged from 62.8 to 68.8 kDa, and the isoelectric point (pI) ranged from 6.29 to 9.05. Notably, the majority of MACPF proteins exhibited basic characteristics, with the exception of CaMACPF1, StMACPF5, and SlMACPF3, which belong to Group III, and had acidic pI values below 7.0.

### 2.2. Phylogenetic and Structure Analysis

**Figure 1:**
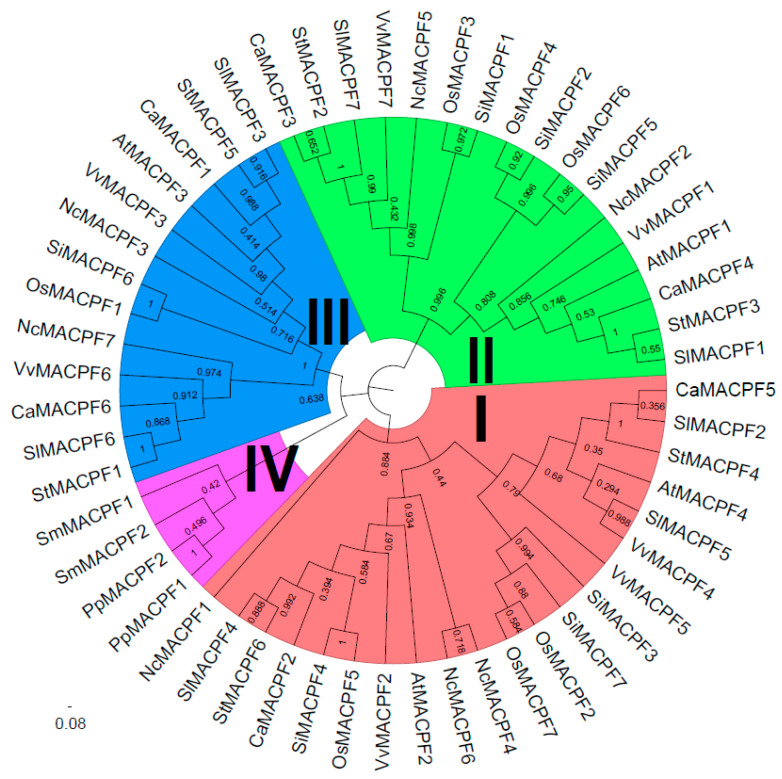
Plant MACPF proteins’ phylogenetic tree analysis. The phylogenetic tree of 55 MACPF proteins’ catalytic domain, including Solanaceae MACPF proteins, was constructed with the MEGA X software, using the Maximum Likelihood method and JTT matrix-based model. Based on the tree, the MACPF proteins were categorized into four distinct groups, represented by four colors, with optimization using FigTree software (v1.4.4.).

To discover the phylogenetic relationship of the selected plant *MACPF* family, the MACPF proteins’ catalytic domain, including Solanaceae MACPF proteins, was constructed using the MEGA X software. The Maximum Likelihood method and JTT matrix-based model were employed for this analysis. Based on the phylogenetic tree and the organization of domains or motifs, the MACPF proteins were classified into four distinct groups (Groups I–IV), with bootstrap values of 88, 99, 100, and 42, respectively (Figure 1). The bootstrap values among the groups were lower than those within the groups, indicating the reliability of the classification. Furthermore, the analysis of the phylogenetic tree and domain organization revealed the presence of characteristic *MACPF* genes in bryophyte and lycophyte lineages, which formed Group IV (Figure 1).

Furthermore, it was observed that the Solanaceae *MACPF* genes were present in three additional groups, namely Group I, Group II, and Group III, with seven, six, and six genes, respectively. Apart from the three *MACPF* members identified in tomato from Group I, two other Solanaceae genomes also exhibited two *MACPF* members each in Group I, Group II, and Group III, suggesting the occurrence of an additional gene duplication event in tomato (Figure 1). Notably, no segmental duplication or tandem duplication events of the *MACPF* genes were detected in the Solanaceae, indicating that dispersed duplication played a prominent role in *MACPF* gene expansion in the Solanaceae genomes.

**Figure 2:**
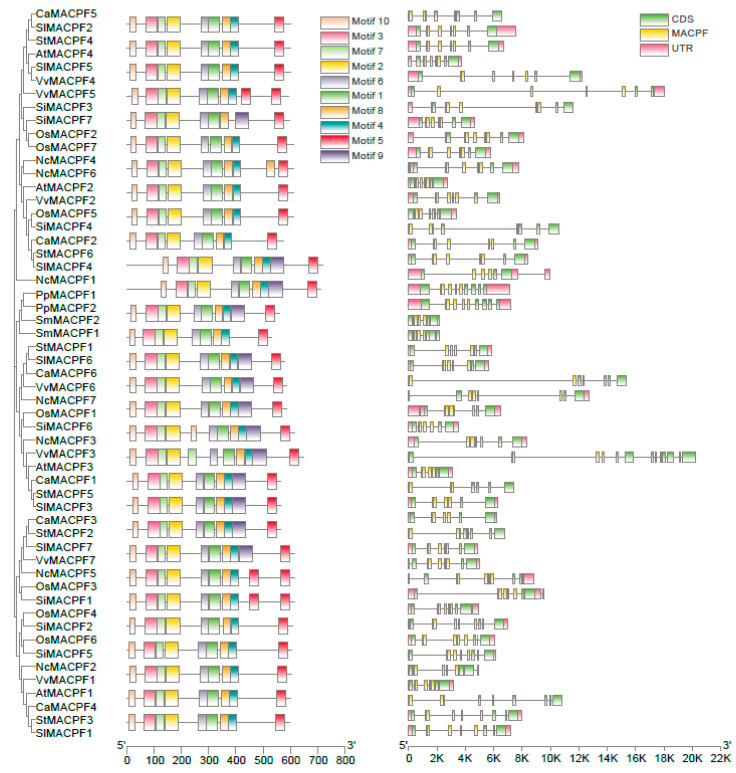
Schematic representation of the conserved motifs and gene structures of plant MACPF. Left panel: a phylogenetic tree redrawn from Figure 1, maintaining the same topology. Middle panel: the compositions of conserved motifs in MACPF proteins, identified using the MEME web tool, with up to 10 conserved motifs. Right panel: the exon–intron structures of the *MACPF* genes, as determined by the GSDS website. Exons and introns are visually represented as filled boxes and grey lines, respectively. The fraction of the gene that encodes the MACPF domain is highlighted in yellow, while the remaining coding sequences are marked in green. The 5′ and 3′ untranslated regions (UTRs) are indicated in red at the ends of the sequences.

The classification, based on the phylogenetic tree, was further validated by the analysis of the MACPF proteins’ domain organization using the multiple EM for motif elicitation (MEME) website. The high E-values observed among the proteins indicates a significant sequence conservation of *MACPF* genes, across plant species, particularly within the Solanaceae family (Figure 2). In order to gain a deeper comprehension of the organization of 55 *MACPF* genes, an exon–intron analysis was conducted using GSDS tools. The *MACPF* genes exhibited a notable resemblance in terms of the exon–intron composition and phase position. Furthermore, the exon–intron gene structures within the subgroups displayed a significant similarity, aligning with the arrangement of protein motifs (Figure 2). The findings revealed that 5′ or 3′ UTR regions of *MACPF* genes, which are recognized as regulatory regions, were predicted in 86% (47/55) of cases, with the exception of spikemoss *SmMACPF1* and *SmMACPF2*, pepper *CaMACPF1/2/4/5/6*, and potato *StMACPF2* (Figure 2), which indicates a greater likelihood of diverse regulatory mechanisms among *MACPF* genes. Moreover, the absence of regulatory untranslated regions in pepper *MACPF* suggests that the evolution of pepper *MACPF* might have occurred earlier.

### 2.3. Solanaceae MACPF Genes Duplication

To determine the physical location and expansion of Solanaceae *MACPF* genes, they were mapped to their respective chromosomes and anchored chromosomes 3, 4, and 4 in St, Sl, and Ca. According to the duplications classification criteria outlined in Reference [25], no instances of tandem duplications were detected in the genomes of the Solanaceae family, which aligns with the evolutionary pattern observed in the Poaceae *MACPF* genes. Furthermore, the Solanaceae genome did not exhibit any documented cases of segmental duplication of *MACPF* genes, which contrasts with the evolution trajectory of the Poaceae *MACPF* genes. The observations suggest that the *MACPF* genes underwent distinct evolutionary processes in monocots and dicots. Moreover, the expansion of the *MACPF* gene family in Solanaceae genomes primarily occurred through dispersed duplications.

**Figure 3:**
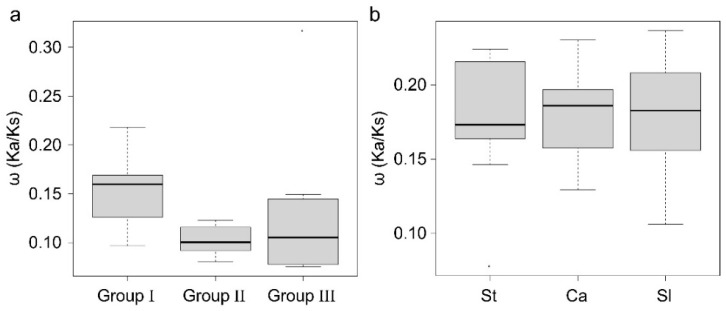
The ω (Ka/Ks ratio) value of *MACPF* genes in Solanaceae. The distribution of ω values was obtained through pairwise comparisons within Groups I, II, and III, (**a**) as well as within Solanaceae species; (**b**) the Y-axis represents the Ka/Ks ratios of *MACPF* genes for each pair. Boxplots were generated in R to illustrate the distribution of these ratio values.

The calculation of the selective pressure upon gene evolution involved the utilization of Ka (non-synonymous distance), Ks (synonymous distance), and ω (Ka/Ks ratio) values [26,27]. According to the neutral theory, the ω value indicates positive selection (greater than 1), neutral evolution (equal to 1), purifying selection or negative selection (less than 1) [28]. To assess the potential variations in selection pressure on *MACPF* genes within and between groups and species in Solanaceae, the gene pairs were examined. The results indicate that the Ka/Ks ratio of Solanaceae *MACPF* gene pairs was consistently below 1, suggesting that these genes have undergone purifying selection. Additionally, the ω values for each *MACPF* group were calculated, resulting in average ω values of 0.15, 0.11, and 0.13 for Group I, Group II, and Group III, respectively (Figure 3a). Group I exhibited a higher ω value compared to the other two groups, indicating a greater relaxation of purifying selection in Group I. Conversely, Group II had the smallest ω value, suggesting a stronger susceptibility to functional differentiation in this group than in the other two groups. None of the Ka/Ks ratios exceeded 1, suggesting a varied purifying selection pressure among the three Groups and no evidence of positive selection during the expansion of the *MACPF* gene family in Solanaceae. The Solanaceae genome species, including the pepper, tomato, and potato genomes, exhibited similar Ka/Ks ratios (Figure 3b), indicating a comparable susceptibility to functional differentiation during the evolution of *MACPF* genes.

### 2.4. Collinearity Analysis of Solanaceae MACPF Genes

**Figure 4:**
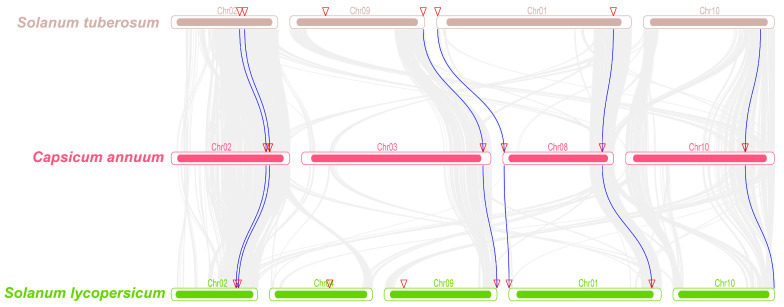
Collinearity analysis of *MACPF* genes among pepper, tomato, and potato. The chromosomes of three Solanaceae species are visually represented as distinct colored boxes. Specifically, the topmost brown boxes correspond to *Solanum tuberosum*, the middle magenta boxes represent *Capsicum annuum*, and the bottom green boxes signify *Solanum lycopersicum*. MCScanX software was employed to establish connections between putative orthologous genes within their genomes’ respective genomes, which are depicted as lines. Collinear relationships among *MACPF* genes are denoted by the innermost grey solid lines. In total, 12 orthologous *MACPF* gene pairs were identified and are connected by blue solid lines.

To further investigate the evolutionary relationships among members of the Solanaceae *MACPF* gene family, the MCScanX software was utilized to identify collinearity relationships among *MACPF* genes across various Solanaceae species. A total of 12 orthologous *MACPF* genes were identified in three Solanaceae genomes, with an e-value of less than 1 × 10^–10^ as determined by the MCScanX software. This analysis revealed six blocks of collinearity between tomato and pepper, as well as six blocks between pepper and potato (Figure 4). Moreover, all these orthologous *MACPF* genes displayed a one-to-one correspondence, indicating a higher level of conservatism among *MACPF* genes across Solanaceae species.

### 2.5. Cis-Regulatory Elements Analysis in Solanaceae MACPF Promoters

**Figure 5:**
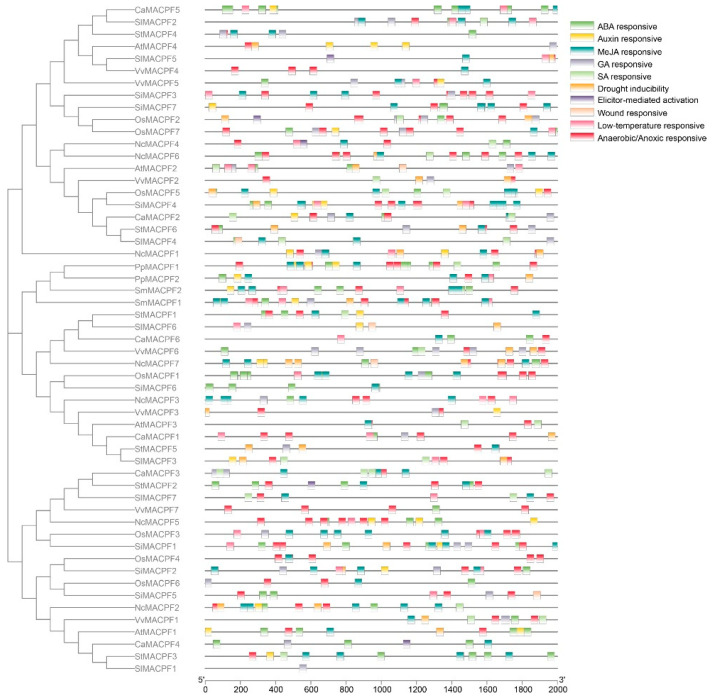
Prediction and analysis of *cis*-regulatory elements (CREs) within the promoter sequences of the *MACPF* gene family. The left side of the figure displays a phylogenetic tree, which has been reproduced from Figure 1. On the right side, the PlantCare database was utilized to predict the CREs within the 2000 bp upstream regions of the 55 *MACPF* genes. These CREs can be classified into two distinct categories: phytohormone-responsive elements, including ABA, Auxin, GA, MeJA, and SA, and stress-related elements, encompassing drought inducibility, low-temperature responsiveness, elicitor-mediated activation, and wound responsiveness.

According to previous studies, it has been observed that plant *MACPF* genes take part in the stress response of Arabidopsis and Poaceae species [2,19,23]. To investigate the potential roles of *MACPF* genes in response to biotic and abiotic stresses, the *cis*-regulatory elements (CREs) of the promoters were analyzed. Specifically, the 2 kb sequence upstream of the *MACPF* gene was examined using the PlantCARE website. The focus was on identifying and characterizing the *cis*-elements of the gene promoters, particularly those related to phytohormones and stress responses, excluding light-responsive elements (Figure 5). A total of 196 potential CREs were identified across the 19 *MACPF* promoters, with 111 CREs found in the promoters of 7 *NcMACPF* genes (Figure 5).

We discovered a total of 44 putative ABA-responsive elements (ABREs) in the promoters of 14 Solanaceae *MACPF* genes (Figure 5), which have been shown to respond to both salt and osmotic stress [29]. In addition, we identified nine elements that are potentially involved in drought stress responses in the promoters of six *SolMACPF* genes. Additionally, we have found 11 putative low-temperature responsive elements (LTREs) in the promoters of eight Solanaceae *MACPF* genes (Figure 5), indicating their potential role in cold stress. Moreover, two putative elements associated with wound responsiveness have been identified in the promoters of two *SlMACPF* genes. Furthermore, our analysis revealed that a subset of Solanaceae *MACPF* genes might be associated with anoxia responses, as evidenced by the presence of gibberellin-responsive (GARE) motifs in 10 Solanaceae *MACPF* promoters. Additionally, 14 Solanaceae *MACPF* promoters contained 28 putative anaerobic- or anoxic-related motifs (ARE and GC-motif), suggesting their involvement in anaerobic or low oxygen stress (Figure 5).

Moreover, we identified several elements, such as the AT-rich motif (TAAAATACT) in *CaMACPF4*, *StMACPF2,* and *SlMACPF5* (Figure 5), which were implicated in elicitor-mediated activation of plant defenses against biotic stress. In our study, we identified 15 SA-Responsive Element (SARE) motifs associated with SA responses in the promoters of 20 Solanaceae *MACPF* genes. Additionally, we found eight auxin response element (ARE) motifs believed to mediate Auxin responsiveness in eight Solanaceae *MACPF* genes (Figure 5). Furthermore, the promoters of 15 Solanaceae *MACPF* genes contained 60 putative TGACG or CGTCA motifs, which were known to be involved in methyl jasmonate (MeJA) responses (Figure 5). These findings provide compelling evidence that Solanaceae *MACPF* genes are likely involved in multiple stress responses.

We conducted further analysis on the promoters of *NcMACPF* genes, wherein we identified a total of 15 putative ABREs in six *NcMACPF* promoters and nine elements associated with drought inducibility in four *NcMACPF* promoters. Additionally, we observed the presence of five putative LTREs in four *NcMACPF* promoters (Figure 5). Notably, only one CRE mediating wound response was identified in *NcMACPF7*, indicating an early functional differentiation (Figure 5). Similar to Solanaceae, a subset of *NcMACPF* genes might exhibit a response to anoxia, as evidenced by the presence of 2 putative GARE motifs in 2 *NcMACPF* promoters, as well as 27 putative ARE and GC-motif elements distributed across all 7 *NcMACPF* promoters (Figure 5).

CREs associated with biotic stress can be detected in *NcMACPF* genes, including one element component believed to be accountable for triggering plant defense activation in *NcMACPF4*. Additionally, there were eight putative AREs in the promoters of four *NcMACPF* genes and three putative SAREs in the promoters of three *NcMACPF* genes (Figure 5). Furthermore, 40 putative MeJA-responsive elements can be identified in the promoters of 6 *NcMACPF* genes (Figure 5). These findings indicated that *NcMACPF*, similar to Solanaceae, exhibits responsiveness to diverse stress stimuli.

### 2.6. Expression Profile Analysis of Solanaceae MACPF Genes

**Figure 6:**
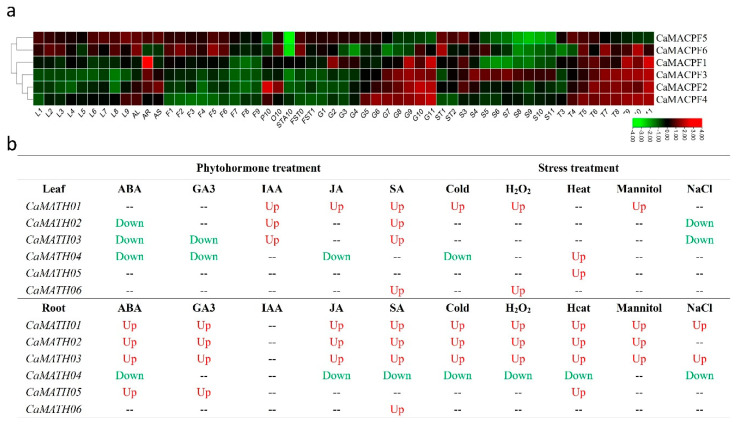
Expression profile of six *CaMCAPF* genes. (**a**) Expression profiles of six *CaMACPF* genes in plant developmental stages. The expression values of the *CaMACPF* genes were resolved from the Pepperhub database (http://www.hnivr.org/pepperhub, accessed on 18 August 2023). L: leaf; F: flower; P: Petal; O: Ovary; STA: Anther; FST: Whole Fruit; G: Pericarp; T: Placenta; ST: Placenta and Seed; S: Seed. The expression values were clustered using heatmap methods. The color bar represents the Z score of the expression value; (**b**) *CaMATEs* regulation upon phytohormone and stress treatments. L: leaves; R: roots; A: ABA treated; S: SA treated; J: JA treated; I: IAA treated; G: GA treated; F: freezing treated; R: H_2_O_2_ treated; N: NaCl treated; M: mannitol treated; H: heat treated; Up (Red): upregulated genes; Down (Green): downregulated genes; --: no regulation.

In order to gain a deeper understanding of the roles played by Solanaceae *MACPF* genes in various stages of development, we collected and analyzed RNA sequencing data from the PepperHub database and Gene Expression Omnibus (GEO) database [30], following the methodology described in a previous publication [31]. The expression values of the 6 pepper *CaMACPF* genes in 57 tissues were resolved and clustered with the value of Reads Per Kilobase of the transcript, per Million mapped reads (RPKM), with visualization using the heatmap method (Figure 6). The analysis revealed that all 6 *CaMACPF* genes were expressed in all 57 pepper tissues (Figure 6). According to the Tissue specificity index (TAU) values, it was observed that *CaMACPF1* exhibited preferential expression in T11 (Placenta) and *CaMACPF6* exhibited preferential expression in T10 (Placenta) with Group III. Additionally, *CaMACPF2* exhibited preferential expression in P10, and *CaMACPF5* exhibited preferential expression in L9 (leaf) within Group I. Furthermore, *CaMACPF3* exhibited preferential expression in T11 (Placenta), and *CaMACPF4* exhibited preferential expression in G11 (Pericarp) within Group II.

The *CaMACPF* within the same group exhibited a discernible expression and regulation pattern in various organs of pepper (Figure 6a), which suggests the occurrence of sub-functionalization or functional diversification among the *CaMACPF* paralogs. Notably, our findings indicate that the majority of *CaMACPF* genes (five out of six) exhibited the highest expression levels in reproductive tissues compared to vegetative tissues, suggesting their significant involvement in plant reproductive organs and tissues. Furthermore, Group I displayed a higher expression pattern compared to Group II and III, as illustrated in Figure 6a.

### 2.7. Expression Analysis of Phytohormone- and Stress-Responsive Pepper MACPF Genes

The experimental elucidation of *MACPF* genes has revealed their significant role in plant response to stress [23]. In order to investigate the function of pepper *MACPF* genes in plant stress response, the expression values of *CaMACPF* genes were obtained from the PepperHub database. The database includes data on pepper roots and shoots subjected to five phytohormones (ABA, GA, indole-3-acetic acid (IAA), JA, and SA) and five stress treatments (freezing, H_2_O_2_, salt, mannitol, and heat stress) [30]. Among the phytohormones, the majority of *CaMACPF* genes in pepper roots were found to be induced by at least one phytohormone, with the exception of *CaMACPF4* repressed in SA, JA, and ABA treatments (Figure 6b).

In both the pepper root and leaves, the expression of *CaMACPF* genes was influenced by various phytohormones. Specifically, *CaMACPF1/2/3* genes were induced by all five phytohormones (ABA, GA, IAA, JA, and SA) in the roots. Additionally, *CaMACPF5* was induced by GA3 and ABA, while *CaMACPF6* was induced by SA in the root. In contrast, in the pepper leaves, a greater number of *MACPF* genes were repressed rather than induced by phytohormones, exhibiting a regulation pattern opposite to that observed in the roots. Specifically, *CaMACPF1* was induced by SA and JA, *CaMACPF2* was induced by SA and repressed by ABA, *CaMACPF3* was induced by SA and repressed by GA and ABA, *CaMACPF4* was repressed by JA and ABA, and *CaMACPF6* was induced by SA. In particular, the presence of the *CaMACPF* gene regulated by IAA was not observed, and *OsMACPF5* was not regulated by any phytohormone (Figure 6b).

In the root tissues, *CaMACPF4* was found to be repressed by all five stress treatments (freezing, H_2_O_2_, salt, mannitol, and heat stress). Conversely, *CaMACPF1* and *CaMACPF3* were induced by all five stressors. *CaMACPF2* showed induction in response to four stressors, excluding NaCl. *CaMACPF5* was only induced by heat in both root and leaf tissues. In the leaf tissues, *CaMACPF1* was induced by mannitol and H_2_O_2_, while *CaMACPF2* and *CaMACPF3* were solely repressed by NaCl. *CaMACPF4* exhibited induction by heat stress but repression by cold stress, and *CaMACPF6* was induced by H_2_O_2_.

Above all, distinct *CaMACPF* gene profiles were observed in roots and leaves in response to phytohormones and stresses, implying the existence of tissue-specific regulatory mechanisms. The involvement of pepper *MACPF* genes, particularly *CaMACPF1/2/3*, in the response to freezing, H_2_O_2_, salt, mannitol, and heat stresses, suggests their significant roles in stress adaptation. Notably, a parallel response pattern was observed between phytohormones and stresses, indicating a shared regulatory pathway in plant stress response.

### 2.8. Pepper MACPF Genes in Response to Submergence

**Figure 7:**
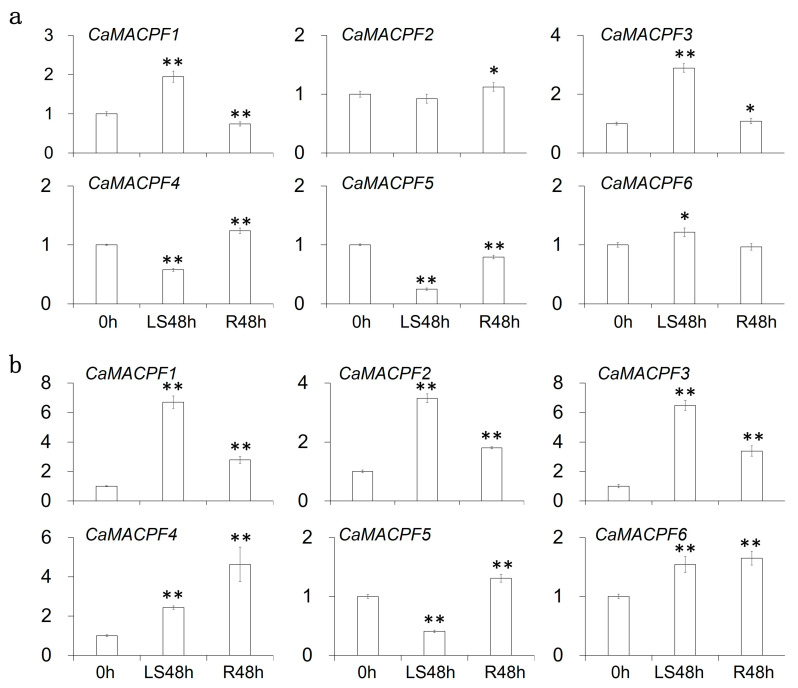
Expression profile of *SolMACPF* genes under submergence treatment. qRT-PCR analysis of *CaMACPF1–6* genes after 48 h of submergence. (**a**) The relative expression of *CaMACPF* genes in pepper roots after submergence for 48 h; (**b**) Relative expression of *CaMACPF* genes in the leaves after 48 h of submergence was assessed. The plants were cultivated in a greenhouse with a temperature of 28/23 °C (light/dark) and a light period of 10 h followed by 14 h of darkness. Transcript levels were normalized using *CaUBI* as the internal reference. Each data point represents the average of three biological repeats. * *p* < 0.05; ** *p* < 0.01 by Student’s *t*-test.

To further investigate the impact of hypoxia stress on the function of pepper *MACPF* genes, plants at the age of four weeks were submerged for a period of 48 h, followed by a recovery period of 48 h. The reverse-transcription and quantitative PCR (qRT-PCR) method was utilized to examine the regulation pattern of six *CaMACPF* genes. The transcription levels of *CaMACPF1*/3 genes exhibited an increase of more than two-fold after 48 h of submergence in the roots of pepper (Figure 7). Additionally, *CaMACPF1/2/3* genes displayed an increase of more than two-fold after 48 h of submergence, while the transcription level of the *CaMACPF4* gene also exhibited an increase of more than two-fold after 48 h of submergence in the leaves of pepper (Figure 7). These results indicated that the induction of *CaMACPF* genes by submergence suggested their potential involvement in the plant’s response to hypoxia and subsequent recovery.

### 2.9. Subcellular Localization of Selected CaMACPF

**Figure 8:**
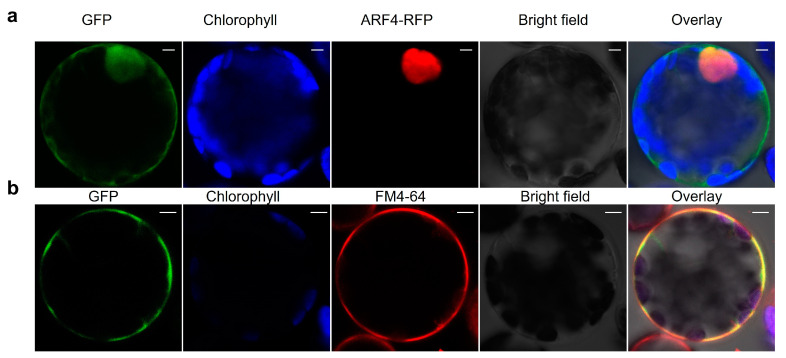
Subcellular localization of CaMACPF6. (**a**) The expression of CaMACPF6-GFP and the nucleus marker ARF4-mCherry, as well as merged images, were also observed; (**b**) The expression of CaMACPF6-GFP and dye FM4-64, as well as merged images, were also observed. The fluorescence signal of CaMACPF6 was predominantly localized in the nucleus and plasma membrane. Bar, 5 μm.

The subcellular localization of pepper CaMACPF6 proteins was investigated through the utilization of a confocal microscope. In order to analyze their subcellular localization, we employed the heterologous expression of CaMACPF6-GFP fusion proteins in Arabidopsis protoplast. The findings indicate that the CaMACPF6-GFP signal predominantly overlapped with the nucleus subcellular marker ARF4-RFP (Figure 8a), as well as with the dye FM4-64 (Figure 8b), which suggests that the CaMACPF6 protein is localized within the nucleus and plasma membrane.

## 3. Discussion

MACPF proteins have been found to have a substantial impact on plant development and response to various stresses, particularly in relation to pathogens and pests affecting both plants and animals [23,32,33,34]. Thus far, the identification of *MACPF* genes in the genomes of Arabidopsis, cotton, and Poaceae species has been accomplished [2,15,35]. However, no *MACPF* genes have been characterized in Solanaceae, a plant family that encompasses several crucial agricultural and horticultural crops, such as tomato, potato, and pepper. In this study, we aim to identify Solanaceae *MACPF* genes and analyze their evolutionary characteristics and functional roles in development and stress response.

### 3.1. The Solanaceae MACPF Genes Conservation in Eudicot and Monocot

Several *MACPF* genes have been identified in Arabidopsis, cotton, and Poaceae species, at the genome level [2,19,23]. Our previous study demonstrated that the occurrence of *MACPF* genes was first observed in the moss *P. patens* but not in algae, suggesting their association with the colonization of land by plants and their classification as a land plant-specific gene family [2]. Furthermore, only Group IV *MACPF* genes were found in the moss and spikemoss, with no other groups identified. In this study, employing similar methods [2], we have identified seven, six, seven, and six *MACPF* genes in *Nymphaea colorata*, *Capsicum annuum*, *Solanum lycopersicum*, and *Solanum tuberosum*, respectively (Table S1, Figure 1). These *MACPF* genes were found to be distributed in Groups I, II, and III, indicating that these three *MACPF* groups likely originated in the basal group of seed plants. Conversely, no Group IV *MACPF* genes were identified in *Nymphaea colorata* and Solanaceae species, suggesting that Group IV is specific to the moss and spikemoss lineage. Moreover, there were at least two members in Groups I, II, and III, suggesting that they might have undergone similar evolutionary processes in the Solanaceae genomes. Notably, the number of members in Groups I and II in Solanaceae genomes was smaller compared to Poaceae genomes, while there were more members in Group III. This discrepancy suggests the presence of distinct evolutionary selectivity patterns between eudicot and monocot.

The parameters Ka, Ks, and ω (Ka/Ks ratio) were utilized to evaluate the selective pressure exerted on gene evolution [26,27]. The ω values derived from pairwise comparisons within groups or species were consistently below 1.0 (Figure 3), indicating that Solanaceae *MACPF* genes underwent purifying selection throughout their evolutionary history, a pattern similar to that observed in Poaceae *MACPF* genes [2]. In contrast to the Poaceae *MACPF* genes, the ω values were significantly smaller in Solanaceae species when comparing across species (Figure 3b), implying that *SolMACPF* genes experienced a greater degree of purifying selection in comparison to Poaceae *MACPF* genes. Additionally, the ω values of Solanaceae *MACPF* genes were lower than those of Poaceae *MACPF* genes in both Groups II and III, with an increase in Group I [2], implying that *SolMACPF* genes experienced a higher level of purifying selection in Groups II and III, but a lesser degree in Group I.

The findings of our study indicate that *SolMACPF* genes could be classified into three distinct groups in angiosperm, as evidenced by the high bootstrap values of phylogenetic trees, similarities in gene structures, arrangement of domains or motifs, and physical and chemical properties (Figure 1 and Figure 2). This classification aligns with previous research on Poaceae *MACPF* genes, which also demonstrated the same grouping as observed in Solanaceae [2]. Furthermore, this classification is consistent in water lily genomes, suggesting a higher degree of conservation of *MACPF* genes among angiosperms.

### 3.2. Solanaceae MACPF Duplications and Collinearity Analysis

In the three Solanaceae genomes, a total of 19 *MACPF* genes were identified, exhibiting an uneven distribution across the chromosomes, which distribution pattern was also found to be similar to that observed in Poaceae genomes [2], but differed from the outcomes of diploidization or polyploidization events [36,37,38]. No clustering of *MACPF* genes was observed in the genomes of the Solanaceae family (Figure 4), which was consistent with the expansion of *MACPF* genes in the Poaceae family [2], suggesting the absence of tandem duplication in the Solanaceae family, based on chromosomal location analysis. Furthermore, unlike the expansion of *MACPF* genes in the Poaceae family [2], no segmental duplications were detected in the *SolMACPF* genes, indicating a divergence between monocotyledons and dicotyledons. Additionally, it was observed that *MACPF* genes in the Solanaceae family did not undergo segmental duplication events, unlike in Poaceae species [2], suggesting a distinct duplication trajectory between eudicot and monocot species. The analysis of three Solanaceae genomes duplication demonstrated the absence of tandem or segmental duplications in the *MACPF* gene, indicating that the expansion of Solanaceae *MACPF* genes was solely attributed to dispersed duplications.

The paralogous *MACPF* genes identified by collinearity analysis in Solanaceae exhibited similar domain organization and gene structures, particularly within the same groups, as evidenced by the significantly higher bootstrap values in the phylogenetic tree (Figure 1 and Figure 2), implying that there were no instances of domain or motif gain or loss during the evolution of *MACPF* genes. In comparison to Poaceae *MACPF* genes, Group III members in the Solanaceae species exhibited an additional duplication, highlighting the distinctiveness of this particular group.

After gene duplication, the duplicated genes might undergo various outcomes, such as sub-functionalization, neo-functionalization, or non-functionalization [39]. The distinct response to phytohormone and stress treatments exhibited by *CaMACPF2* and *CaMACPF5* in Group I, *CaMACPF3* and *CaMACPF4* in Group II, and *CaMACPF1* and *CaMACPF6* in Group III suggests functional divergence or sub-functionalization of duplicated genes following the expansion.

### 3.3. Solanaceae MACPF Genes Function

Previous studies have highlighted the significant roles of plant *MACPF* gene development and stress response [2,15,18,19,22,23,35,40]. The utilization of expression patterns was employed to evaluate the involvement of genes in plant reactions to both biotic and abiotic stresses [41] in conjunction with physiological and biochemical indicators. Analysis of tissue expression revealed that five out of the six *CaMACPF* genes exhibited a preference for expression in the placenta or pericarp, which belong to vegetative tissues, while *CaMACPF5* was predominantly expressed in leaf tissue (Figure 6). This observation suggests that *MACPF* genes in the Solanaceae family might possess significant functions in plant development, particularly during vegetative stages, similar to *MACPF* genes in the Poaceae family [2].

Furthermore, an in-silico analysis of the promoter of the 19 *SolMACPF* genes revealed that the presence of 196 potential CREs associated with phytohormone and stress responses (Figure 5), indicating their involvement in plant adaptation to environmental stimuli. Notably, the examination of RNA-seq expression data for pepper *MACPF* genes corroborated the association between phytohormone and stress response, particularly in the roots (Figure 6b), which aligned with the identification of CRE motifs in the gene promoters. Additionally, our research findings demonstrated that six *CaMACPF* genes exhibited regulation by at least one phytohormone or stress treatment (Figure 6b), suggesting their involvement in complex or interconnected functions in response to environmental stimuli in the Solanaceae family. Particularly noteworthy, our results have revealed that *CaMACPF1/2/3/4/6* were all induced by hypoxia stress, with the exception of *CaMACPF5*, which provided a foundation for further experimental investigation.

## 4. Materials and Methods

### 4.1. MACPF Genes Identificaiton in Solanaceae

To comprehensively analyze the complete genome of the *MACPF* gene family, a Basic Local Alignment Tool for Protein (BLASTP) analysis was conducted. This analysis utilized Arabidopsis and six Poaceae species *MACPF* genes [2,15] as queries, which were against selected Solanaceae genomes, with parameters of the E-value less than 10^–5^, including pepper (*Capsicum annuum*), tomato (*Solanum lycopersicum*), and potato (*Solanum tuberosum*) [36,37,42], as well as the base group of water lily (*Nymphaea colorata*) [24]. Additionally, the MACPF domain Pfam entry (PF01823) was obtained from the Pfam database [43] and used to serve as a seed to identify the *MACPF* gene, through the HMMER 3.0 software [44], with Ensembl Plants [45] and Phytozome databases [46]. The SMART, CDD, and InterProscan databases were then employed to further screen the candidate MACPF proteins, as previously described in a study [2,31].

### 4.2. MACPF Gene Structure, Domain Organization, and Promoter Analysis

The exon–intron organization of the predicted gene structures was detected by the Gene Structure Display Server (GSDS2.0). The conserved motif in the domain organizations was characterized through the MEME (Multiple EM for motif elicitation) database [47]. The putative *cis*-regulatory elements in the 2000 bp upstream of *MACPF* gene promoters were predicted using the PlantCARE database [48]. The results were visualized by the software of TBtools [49].

### 4.3. MACPF Gene Phylogenetic Analysis

The catalytic domains of MACPF proteins were aligned using ClustalX 2.0 [50], and the resulting alignment was used as input for MEGA X to construct a phylogenetic tree [51]. The maximum likelihood method (ML) was employed with the Jones–Taylor–Thornton (JTT) model and suitable parameters, including Gamma distribution evolutionary rates (+G), pairwise deletions, and 1000 bootstraps, which was visualized using FigTree software.

### 4.4. MACPF Gene Duplication and Synteny Analysis

To identify intragenomic and intergenomic syntenic blocks within the Solanaceae, MCScanX software was utilized with the default parameters [52]. The segmental duplication and tandem duplication events were identified according to the previous study [53]. The result was visualized by the software of TBtools [49].

### 4.5. MACPF Gene Expression Analysis

The tissue-, phytohormone- or stress-specific expression profile of Solanaceae *MACPF* genes was determined by re-analyzing the published pepper RNA-seq transcriptome datasets from PepperHub [30]. Differentially expressed genes with a fold-change greater than 2.0 were subjected to heatmap analysis using R software based on Z scores of gene expression values, as described previously [54,55].

### 4.6. Submergence Treatment and Quantitative RT-PCR

Pepper Capsicum 6421 was cultivated in a greenhouse under the conditions of 25/23 °C (day/night) and a photoperiod of 16 h light/8 h darkness. In light submergence (LS) treatment, four-week-old pepper seedlings were submerged in the water with a depth of 10 cm. After 48 h of submergence followed by 48 h recovery, roots or leave from three individual plant were harvested and pooled as a sample. The expression of *CaMACPF* genes was detected using qRT-PCR (quantitative reverse transcription PCR), as described in previous studies [53,56]. After quantifying the expression levels of each gene in various samples and obtaining three replicated Ct values, the Ct difference between the experimental and control groups and the internal reference gene CaUBI-3 (ΔCt) was calculated. Subsequently, the difference in ΔCt between the experimental group and the control group (ΔΔCt) was determined. Finally, the 2^−ΔΔCt^ method was employed to assess the relative changes in Ct values of the experimental group compared to the control sample. Hypothesis testing was conducted using Student’s *t-*test. The primers used in this study can be found in Table S2, with *CaUBI*-*3* serving as the reference gene.

### 4.7. Subcellular Localization of the CaMACPF6

To determine the subcellular localization of the CaMACPF6 protein, the full-length coding sequence (CDS) was obtained from pepper seedlings using reverse transcription polymerase chain reaction (RT-PCR) with gene-specific primers. The amplified CDS was then ligated into pUC121-XTEN-GFP-HA vectors, which had been digested with BamHI, using specific primers containing the BamHI restriction enzymes (Table S2). The construct pUC121-CaMACPF6-XTEN-GFP-HA enables the synthesis of a fusion protein comprising CaMACPF6. Translation of this fusion protein is initiated at the start codon within the StuI restriction enzyme site, which is positioned before the BamHI site and does not induce any frameshift mutations in the CaMACPF6 protein. Following the transformation of the constructed vectors into Arabidopsis protoplast, the resulting fluorescent signal was visualized using a confocal microscope (LSM880 with Fast airy scan), as previously detailed [57].

## 5. Conclusions

In this study, we conducted a comprehensive characterization of the *MACPF* genes in three Solanaceae genomes, resulting in the classification of 19 *SolMACPF* genes into three distinct groups, according to the phylogenetic tree, gene structure, and organization of domain or motif. The intra-genome analysis demonstrated that dispersed duplications primarily contributed to the expansion of *SolMACPF* genes, as opposed to tandem and segmental duplications. Inter-genome collinearity analysis identified 12 orthologous pairs of the *SolMACPF* gene. Furthermore, based on the ω values, it was determined that purifying selection was the predominant evolutionary driving force behind the expansion of the *SolMACPF* gene family. The expression pattern and subcellular localization of the Solanaceae *MACPF* genes indicate their substantial involvement in plant development and response to environmental stimuli, specifically submergence. These findings offer a comprehensive analysis and fresh perspective on the evolution and function of *SolMACPF* genes, serving as a foundation for future functional identification.

## Data Availability

Not applicable.

## Supplementary Materials

The following supporting information can be downloaded at: https://www.mdpi.com/article/10.3390/ijms241713193/s1.
